# Assessing Security Implications of Genome Editing: Emerging Points From an International Workshop

**DOI:** 10.3389/fbioe.2018.00034

**Published:** 2018-03-28

**Authors:** Robin Fears, Volker ter Meulen

**Affiliations:** ^1^European Academies Science Advisory Council, German National Academy of Sciences Leopoldina, Halle, Germany; ^2^InterAcademy Partnership, Trieste, Italy

**Keywords:** genome editing, security, international workshop, research governance, academies

Genome editing, which includes the deliberate alteration of a selected DNA sequence in a cell using targeted nucleases, is greatly facilitating basic research in the life sciences. In particular, it is contributing significantly to our understanding of biological functions and disease mechanisms. The new genome editing tools are expected to empower innovation for societal applications in human and animal health, agriculture and food systems, and the bioeconomy. As with other tools, there may also be potential for misuse, either inadvertently and associated with biosafety concerns or deliberately and associated with biosecurity concerns.

## Assessment by Academies Worldwide

Because of the rapid development and widespread use of tools such as CRISPR-Cas9 in many countries with various, sometimes divergent regulation and governance of research, international dialog is essential for resolving contentious points and evaluating the implications for ensuring responsible research and innovation. Academies of science and medicine worldwide have already undertaken considerable analysis of the potential benefits and risks of genome editing as part of their broader interests in emerging technologies in the biosciences. For example, in Europe, the European Academies Science Advisory Council (EASAC) published a report (EASAC, 2017) last year providing a broad perspective on multiple genome editing applications, and in the US, the National Academies of Science, Engineering and Medicine (NASEM) have published several comprehensive reports, including on gene drives (NASEM, 2016) and human cell editing (NASEM, 2017).

Recently, in October 2017, EASAC and NASEM, together with the global InterAcademy Partnership (IAP) and the German National Academy of Sciences Leopoldina convened an international workshop of experts in genome editing, security studies, and public policy in Herrenhausen, Germany. This meeting addressed some of the emerging implications, for potential benefits as well as potential misuse, and what might be done to mitigate any potential harm. This workshop was designed to emphasize the pivotal role of transparent and inclusive dialog with stakeholders and the promotion of a research culture that builds trust through responsibility and integrity. Researchers cannot dissociate themselves from the uses of the new knowledge they generate and they must take into consideration the reasonably foreseeable consequences of their activities.

A report of this workshop has now (January 2018) been published (IAP, 2018) and our article here briefly draws attention to some of the key areas covered in detail in the report. Initial workshop sessions explored applications of societal value spanning medicine, plant and animal breeding in agriculture, microbial production, and gene drive systems that might transform an entire population of a selected species. Some of the potential opportunities are listed in Table 1. Participants in the workshop acknowledged the importance of doing more to share good practice in research policy and regulation worldwide to allow the flexibility to manage and enable innovation.

**Table 1:** Examples of points emerging from workshop breakout session discussions.

Application of genome editing	Potential benefits	Potential security concerns
Human cells	Better understanding of disease; new targets and models in drug discovery and development; treatments for single or multiple gene disorders	Off-label use somatic editing for enhancement of individuals (for muscle mass, neurology), e.g., for military purposes; germline modification of future populations

Plants and animals in agriculture	Crops with higher yield, increased nutrient content, resistance to biotic and abiotic stress; improved livestock health, welfare, and productivity	No new categories of risk discerned but research locations may expand beyond traditional management frameworks; relative lack of traceability in edits challenges regulation and enforcement

Gene drive	Control of insect vectors of disease; correcting previous disturbances to vulnerable ecosystems (e.g., reversing rodent invasion of islands)	Potential threats to health and agriculture (although it is assumed there would be easier ways to cause harm); concern that controversy may undermine broader public confidence in science

Microbes	Novel pharmaceuticals, including antibiotics, high-value chemicals, biofuels, biosensors, and other applications in bioeconomy	Similar possibilities to other microbial manipulation methods, e.g., altered pathogens; digitalization of DNA increasingly important in widening access to results

*See IAP (2018) for further detail*.

## Addressing Concerns on Potential Misuse

A main focus of the workshop was to review concerns about misuse, appertaining to the possibilities that the widespread adoption of genome editing might expand research outside of regulated laboratory settings and, wherever located, might also elicit new national security concerns. For example, in the US, security concerns have been expressed by the President’s Council of Advisers on Science and Technology and by the national intelligence community (EASAC, 2017; Fears and ter Meulen, 2017) and NGOs have also inferred (Friends of the Earth, 2017) that government funding of gene drive research denotes military or other national security interests. Although these security alarms lack detail, it is relevant to include consideration of possibilities for misuse when deriving principles for the responsible use of biotechnologies (Wolpe et al., 2017), including genome editing. Academies of science and their networks have been assiduous in these regards (NASEM, 2016, 2017; EASAC, 2017, and see IAP, 2018 for other academy sources). Because some of the security concerns may be application specific and because public anxieties about genome editing—whether relating to safety or security—tend to be about the specific application rather than the technology itself (Gaskell et al., 2017), the workshop was designed also to evaluate concerns in terms of specific sectors, although it became clear that some concerns crossed application boundaries (Table 1).

We emphasize that the focus of the discussions was on “potential.” The science is advancing rapidly but timeframes are uncertain and, indeed, proof of principle for the application has not yet been established in many cases. Therefore, reaching consensus on which, if any, concerns are realistic will be challenging. More robust assessment of the feasibility and probability of concerns is warranted, and there is need for better understanding about the conditions that may repurpose technology for hostile use—evaluating intent as well as accessibility. Moreover, it can be difficult to separate safety from security consequences. Nonetheless, even though there is much more to be done in clarifying the evidence base, the academies through IAP, have already built good connections with policy-makers, particularly in the Biological and Toxins Weapons Convention, to inform about recent scientific developments (IAP, 2017).

There is more to be done to clarify what is new about the issues raised by genome editing, whether these new tools will facilitate outcomes that could already be imagined by other methods, and whether additional risks are conferred. Even if the advent of genome editing were to raise new issues, these should be set into a broader context. First, to appreciate that the success of new tools depends on the opportunities created by the accumulation of other modern biosciences research outputs (particularly those associated with declining costs of gene sequencing and synthesis) so that a much larger accrual of research advances is necessarily implicated in any concerns. Second, to appreciate that the wider use of such tools does not in itself promote intent to nefarious action.

What are the possibilities to prevent or mitigate security issues? When genome editing is viewed in the broader context, it can be seen that there is a wide range of legal, regulatory and policy strategies, norms of responsible behavior and voluntary guidelines, together with educational, scientific, and technical strategies already available to mitigate potential risks. The disparate elements in this framework of protection are discussed in detail in the report (IAP, 2018) and, to note just one of these elements, it is crucial for the scientific community to share and implement good practice in self-regulation. The German National Academy of Sciences Leopoldina has worked with scientific partners to develop model rules (DFG and Leopoldina, 2016) on scientific freedom and responsibility in handling security-relevant research. Committing to self-regulation, while minimizing bureaucracy, helps to address a common concern within the scientific community that additional governance measures would hamper responsible research without diminishing the likelihood of intentional misuse.

## Public Engagement and Global Coordination

The workshop concluded with a discussion of the next steps required both to ascertain and clarify what is currently uncertain in the evidence base and to communicate about the continuing responsibility of the scientific community to tackle these complex topics. As with other emerging technologies, a lack of communication about uncertainties may undermine public confidence in science. It is vital that younger scientists and researchers worldwide have a voice in the continuing public dialog. Standards of evidence are important. Scientists need to build trust with the security community as well as the public-at-large, recognizing that there are differing perceptions of threats and differing expectations of evidence. There should be balanced and open discussion of the potential benefits and any safety or security issues, particularly as they relate to consumers. In order to resolve uncertainties, the dimensions of security must be well defined: security concerns can apply to public health, food, national economies, data, and privacy, for example, as well as to biological weapons. Many consider that genome editing can itself assist in tackling security challenges, such as for health and food, and help to provide countermeasures.

It is important to develop international coherence in research management to enable innovation (Gaskell et al., 2017), and the workshop discussion emphasized opportunities for global coordination in responsible science guidelines and their monitoring, research standards, risk assessment, and management procedures. Risk assessment and mitigation are intrinsic to all scientific developments. The academy organizers regard this intensive and diverse workshop as a significant first step in an ongoing process. It is deemed highly desirable to develop a sustainable network encompassing the scientific and security communities, and others, to share perspectives, facilitate information exchange, identify priorities for further study, and serve as a basis for extending engagement more widely.

## Author Contributions

RF provided a first draft of this article and of the Herrenhausen report (IAP, 2018). VtM chaired the organizing committee of the Herrenhausen workshop and the earlier EASAC Working Group (EASAC, 2017) and revised this article.

## Conflict of Interest Statement

The authors declare that the research was conducted in the absence of any commercial or financial relationships that could be construed as a potential conflict of interest.
